# Effects of an mHealth Occupational Therapy Intervention on Functional Performance: A Pilot Study

**DOI:** 10.3390/healthcare13162015

**Published:** 2025-08-15

**Authors:** Irene Pérez-Díaz, Mario Arnáiz-González, Estíbaliz Jiménez-Arberas

**Affiliations:** Faculty Padre Ossó, Centre Attached to the University of Oviedo, 33008 Oviedo, Spain

**Keywords:** mHealth, usability, pediatrics, neurodevelopmental disorders

## Abstract

Neurodevelopmental disorders are one of the most prevalent conditions today, and among the limitations in activity and restrictions in the participation of children and their families, we find intervention in activities of daily living; therefore, research focused on outcome measurement is one of the most active lines, and after COVID-19, telerehabilitation has garnered special interest. Background/Objectives: The study objective was to evaluate the effectiveness of a mobile health (mHealth) application in improving the performance of activities of daily living in children with neurodevelopmental disorders. Methods: The study employed a quasi-experimental design with a control group, using a fully remote mHealth-based intervention. The instruments used were a sociodemographic ad hoc, Pediatric Evaluation of Disability Inventory Computer, Family Outcomes Survey, Family Confidence Scale, and System Usability Scale. The final sample consisted of 13 participants. Results: The mHealth intervention showed significant improvements in occupational performance in the experimental group, especially in the global score and in the Responsibility dimension of the PEDI-CAT. No relevant differences were observed in the CON-FAN and FOS scales between groups, although the latter showed improvements over time. The usability of the app was rated positively (SUS = 69.75). Conclusions: The developed application presents good usability for families of children with neurodevelopmental disorders, but to obtain better outcome measures, the intervention should combine face-to-face sessions and the use of mHealth, as well as employing the family-centered model.

## 1. Introduction

Neurodevelopmental disorders (NDDs) encompass a broad and heterogeneous group of conditions that begin in childhood and are directly related to alterations in the development of the central nervous system. According to the Diagnostic and Statistical Manual of Mental Disorders (DSM-5), this category includes diagnoses such as Attention-Deficit/Hyperactivity Disorder (ADHD), Autism Spectrum Disorder (ASD), intellectual disability, communication disorders, motor disorders, specific learning disorder, and various genetic conditions, such as Down syndrome [1]. These conditions can significantly interfere with functional development, social participation, academic performance, and daily life activities of children and their families, posing a multidimensional challenge at clinical, educational, and social levels.

At a global level, the prevalence of neurodevelopmental disorders (NDDs) shows considerable variability, mainly attributed to methodological differences, the diagnostic criteria applied, and the characteristics of the populations studied. According to the review conducted by Francés et al. (2023) [2], international estimates indicate a prevalence of 0.63% for intellectual disability, between 5% and 11% for Attention-Deficit/Hyperactivity Disorder (ADHD), 0.70% and 3% for Autism Spectrum Disorder (ASD), 3% and 10% for specific learning disorders, 1% and 3.42% for communication disorders, and 0.76% and 17% for motor disorders. These figures reflect not only the magnitude of the phenomenon on a global scale but also the heterogeneity with which NDDs manifest across different sociocultural and healthcare contexts, highlighting the urgent need to standardize detection and diagnostic processes. In the Spanish context, the study by Bosch et al. (2022) [3] provides key evidence on the prevalence of NDDs and their sociodemographic correlates in a large sample of school-aged children. Through a structured screening and diagnostic confirmation process, it was found that 18.3% of the evaluated children and adolescents (*n* = 6834) met criteria for at least one neurodevelopmental disorder, although only a portion had received a previous diagnosis. The highest prevalence rates were observed in ADHD (9.92%) and specific learning disorder (10.0%), followed by communication disorders (1.05%), motor disorders (0.76%), ASD (0.70%), and intellectual disability (0.63%).

Children with NDDs frequently face significant challenges in performing activities of daily living (ADLs), including personal hygiene, dressing, feeding, and engaging in play and leisure. These difficulties are not only related to sensory processing and motor planning deficits but also to social-communicative limitations that interfere with autonomy and occupational engagement [4]. Such impairments in daily functioning can persist across developmental stages and often contribute to high levels of caregiver burden and reduced family quality of life [5].

The systematic review by Laverdure and Beisbier (2021) [4] synthesized evidence on occupation- and activity-based interventions for children and youth aged 5 to 21 with various neurodevelopmental disorders, including ASD. Their findings demonstrate that contextualized, client-centered approaches are effective in improving ADL performance and participation. Among evidence-based interventions, the Cognitive Orientation to daily Occupational Performance (CO-OP) approach has been adapted successfully for children with ASD. Although originally developed for Developmental Coordination Disorder, CO-OP has shown promising results in children with ASD by helping them develop cognitive strategies to approach complex tasks. Lucas et al. (2016) [6], in their meta-analysis, report that interventions targeting motor performance—frequently co-occurring areas of difficulty in ASD—also contribute positively to broader functional outcomes. These interventions are particularly effective when they integrate task-oriented, goal-directed training rather than isolated motor exercises, aligning with occupational therapy principles. In addition to the above, it should be mentioned that, as an active field of clinical and research application, the use of virtual technologies in occupational therapy has gained increasing support as a viable method for delivering early intervention services. Martínez-Rico et al. (2024) [7], in a study conducted in Spain, analyzed the feasibility, perceived usefulness, and potential of tele-intervention in early childhood services. Their findings highlighted important factors such as usability, effectiveness, professional competence, and trust, which contribute to what they define as social validity. These results are aligned with other studies that evaluate the role of professionals in validating the implementation of telehealth models. This evidence is consistent with the Occupational Therapy Practice Framework (OTPF-4) [8], which describes virtual interventions as the use of simulated, real-time, or asynchronous technologies—such as telehealth and mHealth—for delivering services without physical contact. These methods include videoconferencing, teleconferencing, and mobile applications to plan, implement, and evaluate occupational therapy interventions, education, and consultation [8]. Integrating these technologies can enhance access, continuity of care, and family involvement in pediatric populations [9].

Therefore, the objective of the present study was to evaluate the effectiveness of a mobile health (mHealth) application in improving the performance of activities of daily living (ADLs) in children with neurodevelopmental disorders. The specific objectives were, first, to evaluate the usability and acceptability of the mHealth application from the perspective of the users.

## 2. Materials and Methods

### 2.1. Design

The study employed a quasi-experimental pre-test–post-test design with a control group, using a fully remote mHealth-based intervention.

### 2.2. Sample

A total of 128 organizations—including associations, special education schools, parent–teacher associations (AMPAs), and non-profit entities—were initially contacted via institutional email. In addition, the website [https://mapeoderecursos.inypemalivinglab.es/ (accessed on 7 August 2025)], which provides a recommended mapping of child-focused resources in Asturias, was consulted. However, due to the limited response rate, contact was extended to national-level institutions.

The initial communication included a brief dossier outlining the objectives of the study and the inclusion and exclusion criteria. Given the low number of responses, follow-up telephone contact was made with 44 institutions, primarily federations encompassing multiple local entities within the same autonomous community.

Ultimately, 14 organizations agreed to collaborate in the dissemination of the study. An information dossier was provided for families, who could express their interest in participating by contacting the respective center. A designated staff member from each center acted as an intermediary to explain the project to interested families.

Following this process, 47 families who met the inclusion criteria expressed interest in the study. After receiving detailed information about the study objectives and methodology, 20 families provided informed consent to participate. These families were randomly assigned to either the experimental group (*n* = 10) or the control group (*n* = 10). During the course of the study, seven participants dropped out—six from the control group and one from the experimental group—primarily due to time constraints and lack of adherence to the intervention protocol.

Inclusion criteria for participation were (a) children between the ages of 3 and 9 years, (b) with a confirmed diagnosis of a neurodevelopmental disorder, and (c) presenting significant limitations in participation in activities of daily living (ADLs). Additionally, participants were required to demonstrate a minimum level of technological familiarity, defined as at least level A2 on the Generation D digital competence framework [10], to ensure their capacity to engage in a fully remote, technology-based intervention. Finally, informed agreement to participate in the study was necessary, including willingness to take part in initial interviews and follow-up assessments. Exclusion criteria for the study included the absence of access to a stable internet connection, which would prevent proper participation in a fully remote intervention. Families who did not demonstrate sufficient engagement in the intervention process—such as lack of feedback or discontinuation of assigned activities—were also excluded. Additionally, children with an extremely low baseline functional level that precluded any form of home-based intervention were not considered eligible. Finally, participants who failed to complete the required pre- or post-intervention assessments were excluded from the final analysis.

### 2.3. Instruments

1.-The Ad Hoc Sociodemographic Questionnaire for Children and Families questionnaire consisted of 43 questions, with 33 pertaining to demographic data and the child’s clinical history and 10 addressing family-related aspects such as parental education levels and knowledge and use of technologies.

2.-The Pediatric Evaluation of Disability Inventory Computer Adaptive Test (PEDI-CAT) is used to measure activity and participation outcomes [11,12] in children aged 0–21 with a variety of diagnoses. It is used to evaluate children’s performance across 4 different domains: (1) Mobility (e.g., ambulation); (2) Daily Activities (e.g., dressing); (3) Social Cognitive (e.g., interaction); and (4) Responsibility (e.g., staying safe).

3.-The Sensory Profile 2 (SP-2) [13] is an assessment tool designed to evaluate children’s sensory processing patterns in the context of everyday life. It is based on the theoretical premise that individual differences in sensory processing can either support or hinder a child’s participation in daily activities. The tool consists of questionnaires completed by parents (or caregivers) and teachers, providing scores across various domains including the child’s sensory systems, behavior, sensory patterns, and school-related factors. These results assist professionals in identifying sensory processing difficulties and in planning effective, individualized interventions. The SP-2 is intended for children and adolescents aged 3 years to 14 years and 11 months.

4.-The Family Outcomes Survey (FOS) [14,15] included 24 items measuring family knowledge and skills across five outcomes: understanding their child’s strengths, needs, and abilities; knowing their rights and advocating for their child; supporting their child’s development and learning; building support systems; and accessing the community. Items were rated on a 5-point Likert scale (nothing, a little, somewhat, almost, completely).

5.-The Family Confidence Scale (Con-Fam SCALE) [15,16] measured family confidence through two subscales: Con-Fam CAN (α = 0.96), assessing caregivers’ perceived confidence in helping the child participate in daily routines, and Con-Fam CAF (α = 0.94), assessing self-perceived confidence in supporting oneself and one’s family. The scales comprised 20 and 18 Likert-type items (1–4), respectively.

6.-The System Usability Scale (SUS) [17,18] is a standardized and widely used tool for assessing the perceived usability of products, systems, or applications, particularly in the field of human–computer interaction. Developed by John Brooke in 1986, the SUS consists of 10 items rated on a 5-point Likert scale, ranging from “strongly disagree” to “strongly agree.” The items alternate between positively and negatively worded statements to minimize response bias. The overall SUS score ranges from 0 to 100, with higher scores indicating better usability. A score above 68 is generally considered indicative of acceptable usability, while scores above 80.3 are associated with excellent usability and strong user satisfaction. The SUS is valued for its simplicity, reliability, and validity across diverse contexts and technologies, including mobile health (mHealth) applications and telehealth platforms. Its concise format makes it suitable for both research and clinical evaluation of user experiences. According to the systematic review by Vlachogianni and Tselios (2021) [19], the SUS is considered one of the most robust and consistent tools for assessing user experience in digital health systems, being applicable in heterogeneous environments such as the one represented in this study. Furthermore, longitudinal research by Bangor, Kortum, and Miller (2008) [17], based on more than 200 studies and a decade of use, demonstrated its psychometric reliability (α = 0.911) and its ability to discriminate between differing levels of perceived usability.

### 2.4. Procedure

The mHealth app is a digital platform designed to support communication and therapeutic follow-up in telerehabilitation processes, particularly aimed at children with neurodevelopmental disorders. The tool includes different user profiles—administrators, professionals, families, and patients—each with specific levels of access. All users can access common features such as a personalized itinerary, chats, announcements, patient history, recommendations, documents, links, and an FAQ section. The professional account offers additional advanced management tools, including scheduling appointments, managing agendas and chat inboxes, and creating and organizing documents in PDF format, useful links, tags, and rehabilitation content. Patient and family accounts are focused on accessing and tracking treatment, with the ability to view clinical history, scheduled appointments, recommendations, and shared rehabilitation materials. The application delivers therapeutic activities and interventions in the form of videos, infographics, and worksheets, all of which are categorized according to the International Classification of Functioning, Disability and Health (ICF) [20,21].

Some of these resources are freely accessible to the public (and can be found on the following platforms: https://www.youtube.com/@InypemaClinicaUniversitaria/playlists (accessed on 7 August 2025) and https://sway.cloud.microsoft/lZgBYGh5ooo5EGoL?ref=Link (accessed on 7 August 2025)). The platform presents all content through a simple and intuitive interface, allowing for centralized therapeutic management and promoting continuity of care in both in-person and remote settings. The steps to carry out the study and recruit participants are detailed below.

1.-Participant recruitment: Educational centers, family associations, and clinics serving children aged 3 to 9 years were contacted via telephone and email to invite participation.

2.-Screening and eligibility: Families completed a sociodemographic questionnaire and a digital competence self-assessment based on the Generación D framework (2024) [10]. Only those meeting the inclusion criteria advanced to the next phase. Participants were assigned to either the experimental group or a waitlist control group, both of which were assessed before and after the intervention. Digital literacy was identified as a key variable influencing engagement with mHealth interventions [22,23] and was therefore considered in group allocation to ensure baseline equivalence, reducing confounding effects more effectively than post-intervention statistical adjustments such as ANCOVA.

3.-Group assignment: Given the small sample size and the potential influence of digital competence on engagement with mHealth interventions, participants were allocated to the experimental or control group using a non-random allocation method based on covariate balancing. Specifically, participants’ scores on a digital competence self-assessment were used to ensure that both groups had comparable mean levels of digital literacy. This covariate balancing approach was chosen to minimize confounding effects and ensure baseline equivalence between groups, as digital competence was considered a key variable influencing adherence and feasibility in remote interventions. No formal randomization was performed.

4.-Initial interview and baseline assessment: Families participated in a video call with an occupational therapist to explore the child’s functional challenges, daily routines, and parental concerns. Together, they established individualized intervention goals. Families received training in the use of the mHealth app and supporting materials. Baseline assessments were conducted using standardized tools (PEDI-CAT, Sensory Profile 2, CON-Fam, and FOS).

5.-Intervention delivery: Over four consecutive weeks, families in the experimental group received a fully remote, individualized intervention. Therapists provided 1–2 tailored resources per week (e.g., infographics, videos, activity sheets) through the mHealth platform, based on each child’s needs. Continuous communication (2–3 times per week) via instant messaging and video call allowed real-time feedback and adaptation of the intervention. To ensure consistent adherence, the study implemented a minimum engagement threshold in terms of content viewed and follow-up interaction. Families who did not meet this threshold were excluded from the final analysis. This strategy resulted in a reduced sample size but ensured that participants retained in the study exhibited high and relatively uniform levels of adherence, minimizing the potential confounding effect of differential engagement.

6.-Post-intervention assessment: The same instruments used at baseline were re-administered to evaluate changes in performance, participation, and family well-being following the intervention.

### 2.5. Ethical Considerations

The Research Ethics Committee for Medicinal Products of the Principality of Asturias approved the project titled “Outcome Measures in Natural Environment Interventions in Early Childhood Intervention” under the code CEImPA 2023.342.

### 2.6. Data Analysis

Since the study design includes an inter-subject factor (group: experimental vs. control) and an intra-subject factor (time: pre- and post-intervention), the analysis used was a two-factor ANOVA with repeated measures on one. This model allows for the simultaneous assessment of the main effects of group and time, as well as their interaction, which is essential to detect whether the evolution of scores differs between groups over time. The AB-CA-MR model requires certain assumptions to be met: (1) independence of observations, guaranteed by independent sampling; (2) normality of scores, assessed with the Shapiro–Wilk test; (3) homogeneity of variances between groups, tested with the Levene test; and (4) multigroup sphericity, although the latter does not strictly apply as it has only two levels in the repeated factor (pre and post), which guarantees sphericity by definition. These conditions ensure the validity of the F-tests used in the analysis. Statistical significance was set at *p* < 0.05. Effect sizes were not calculated, and application usage metrics were not analyzed, although they were recorded.

## 3. Results

The study sample comprised ten boys and five girls, aged between 36 and 108 months (M = 87.6, SD = 26.4), all residing in Spain. The mean age of participants in the experimental group was 89.33 months (SD = 22.78), while the control group had a mean age of 84.40 months (SD = 34.83). All participants had a formal diagnosis of a neurodevelopmental disorder: Autism Spectrum Disorder (*n* = 9), Down syndrome (*n* = 4), Angelman syndrome (*n* = 1), and Attention-Deficit/Hyperactivity Disorder (ADHD) (*n* = 1).

With regard to the Sensory Profile, the results for each quadrant are presented descriptively below (see Table 1). This instrument was used to guide the intervention carried out by the occupational therapist.

The data met the assumptions of normality in all subgroups analyzed, as all Shapiro–Wilk tests were non-significant. Firstly, the descriptive results of the PEDI-CAT scale are presented (see Table 2).

**Figure 1:**
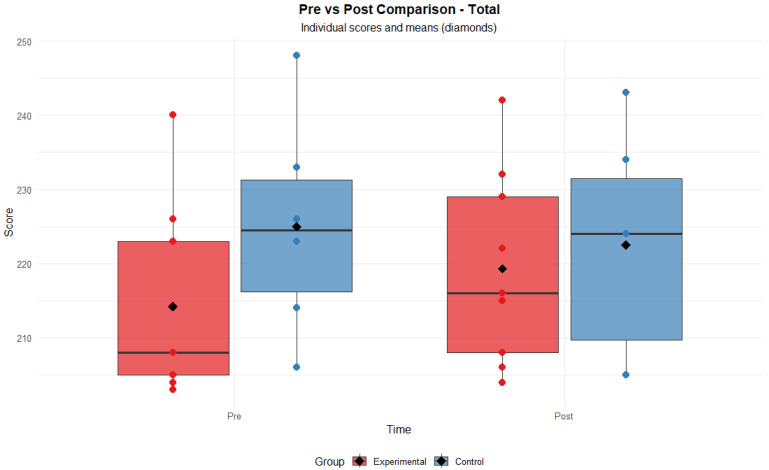
Global PEDI-CAT total pre- and post-evaluation scores.

Furthermore, homogeneity of variances was confirmed by Levene’s test for the Responsibility dimension (*p* = 0.91) and the overall score (*p* = 0.99). Therefore, it is considered that the mixed ANOVA model applied is adequate for the analysis of the data obtained. No significant effects of group (F = 0.95, *p* = 0.347) or time (F = 1.11, *p* = 0.312) were found for the overall PEDI-CAT score, but there was a significant interaction between the two factors (F = 9.4, *p* = 0.009). This result suggests that the intervention produced differentiated changes depending on the assigned group, reinforcing the hypothesis of an effect attributable to the mHealth intervention (see Figure 1).

**Figure 2:**
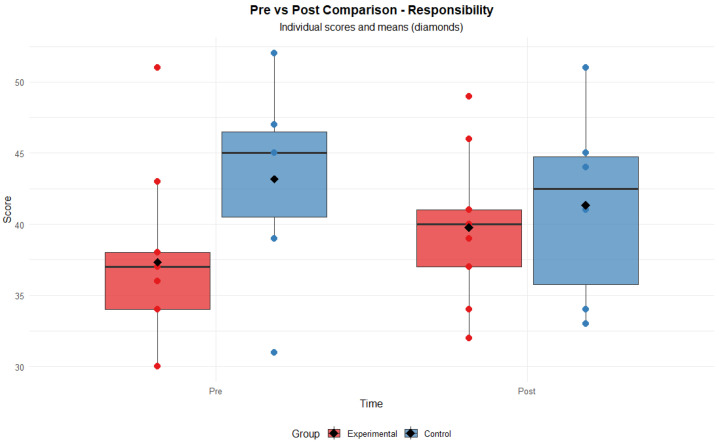
PEDI-CAT pre- and post-assessment scores for the PEDI-CAT Responsibility domain.

It should be noted that in the Responsibility dimension of the PEDI-CAT, no significant main effects of group (F = 1.29, *p* = 0.276) or time (F = 0.11, *p* = 0.747) were observed. However, a significant interaction between group and time was detected (F = 5.32, *p* = 0.038), indicating that the evolution of scores over time differed between participants in the experimental and control groups (see Figure 2).

Secondly, the results obtained on the CON-FAN scale are presented. The ANOVA revealed a statistically significant intercept (F(1, 13) = 246.68, *p* < 0.001, ges = 0.947), indicating that overall scores were significantly different from zero; however, this parameter is typically not interpreted further. The main effect of group was not statistically significant (F(1, 13) = 1.98, *p* = 0.182, ges = 0.127), suggesting no significant differences between the experimental and control groups across time. Although the generalized effect size was moderate, the result did not reach statistical significance. The main effect of time approached significance but did not reach the conventional threshold (F(1, 13) = 3.17, *p* = 0.098, ges = 0.012). This result indicates a trend toward change from pre- to post-test across both groups, with a small effect size. Finally, the group × time interaction was not significant (F(1, 13) = 0.03, *p* = 0.866, ges ≈ 0.0001), indicating that the pattern of change over time did not differ between the experimental and control groups. The interaction effect size was negligible.

Thirdly, the results obtained on the FOS scale. The ANOVA showed a significant intercept (F(1, 13) = 366.92, *p* < 0.001, ges = 0.9643), indicating that the overall mean was significantly different from zero. As expected, the effect size for the intercept was very large. The main effect of group was not statistically significant (F(1, 13) = 1.564, *p* = 0.233, ges = 0.1032), suggesting no significant differences between the groups. The effect size was small to moderate. A significant main effect of time was observed (F(1, 13) = 6.04, *p* = 0.029, ges = 0.0200), indicating that scores changed significantly across time points. However, the effect size was small. The group × time interaction was not significant (F(1, 13) = 0.057, *p* = 0.816, ges = 0.0002), suggesting that both groups followed a similar pattern of change over time. The effect size was negligible.

### Post Hoc Power Analysis and Effect Sizes

A comprehensive post hoc power analysis was conducted to evaluate the statistical power of the repeated measures ANOVA for detecting group × time interactions. The analysis revealed that with our sample sizes (*n* = 8 experimental, *n* = 7 control), the study had limited statistical power ranging from 5.7% to 8.6% across dimensions to detect the observed small to small–medium effect sizes.

Effect sizes were calculated using Cohen’s f and generalized eta-squared (ges) for all group × time interactions: DL dimension: F(1,13) = 1.67, *p* = 0.219, f = 0.096, ges = 0.009; Mobility dimension: F(1,13) = 1.31, *p* = 0.273, f = 0.081, ges = 0.006; Social dimension: F(1,13) = 1.02, *p* = 0.330, f = 0.087, ges = 0.007; Responsibility dimension: F(1,13) = 5.32, *p* = 0.038, f = 0.175, ges = 0.030*; and Total score: F(1,13) = 9.40, *p* = 0.009, f = 0.146, ges = 0.021.

The sensitivity analysis indicated that with the current sample size, only large effect sizes (f ≥ 0.898) could be detected with 80% statistical power. To achieve adequate power (80%) for the observed effect sizes, sample sizes ranging from 129 to 602 participants per group would be required, depending on the dimension analyzed.

The usability evaluation using the SUS scale yielded an average score of 69.75. According to the reference values proposed by Bangor et al. (2008) [17], this value is close to the average of the adjective category “Good” (72.75), suggesting a generally positive perception of the application.

Furthermore, according to the same study, this score corresponds to the third quartile, indicating that the perceived usability is above the overall average. This interpretation is supported by the high correlation between SUS scores and adjective labels (r = 0.806), which validates the use of these categories as an additional interpretative criterion.

## 4. Discussion

The results obtained in this study indicate that the adapted mHealth intervention showed a differential impact on certain dimensions assessed in families with children with neurodevelopmental disorders. The sensory processing patterns observed in this study reveal a heterogeneous profile among children with neurodevelopmental disorders. Descriptive data from the Sensory Profile quadrants highlight significant differences, particularly in the domains of registration and sensitivity, which presented the widest variability. These findings align with Dunn’s Sensory Processing Framework [13], which conceptualizes Sensory Profiles based on the interaction between neurological thresholds and self-regulation strategies. More precisely, significant group–time interactions were observed in the “Responsibility” dimension and in the overall score derived from the PEDI-CAT, suggesting that the experimental group experienced positive changes over time, in contrast to the control group. These findings support the hypothesis that personalized digital interventions can contribute to functional improvements in the family context. Such interventions may be effective not only because of their impact on the intervention with users, but also because they offer a number of benefits such as reducing rehabilitation costs, increasing motivation and participation by users and their families, and facilitating intervention in the environment [24,25].

These results are consistent with the previous literature showing how family-centered models and practice are effective for early intervention with children as well as for preventive pediatric intervention. In this way, the child’s family members are actively involved in the active participation in the daily life of the youngest children. Through a professional-led intervention, developed in the environment by the family members and implemented with the participation of the child, better results are achieved in the evolution of the children [26]. In addition, the present app has been created from scratch and with specific content for use by families, with simple language and visual support and continuous guidance. The use of mHealth can be a great tool for transfer to the child’s natural environment and a support guide for families [27]. This study supports prior findings on the relevance of family quality of life (FQoL) in the rehabilitation of children with neurodevelopmental disorders. Telerehabilitation has been associated with reduced logistical strain and greater parental involvement [9]. However, its suitability may be limited in cases with complex sensory or behavioral needs. Technological barriers—such as poor internet access, low digital literacy, and lack of private space—can compromise intervention effectiveness [28]. A potential challenge in the use of telerehabilitation for children with neurodevelopmental disorders is participant dropout, which can affect the continuity and impact of the intervention. In the present study, which was conducted entirely through telerehabilitation, 5 out of 20 families discontinued their participation, resulting in a 35% dropout rate. This is consistent with previous findings, where dropout rates in similar interventions ranged from 0% to 54%, with a median of around 15% in computer-based programs [12]. Likewise, research in Tehran reported a 19.3% dropout over six months, influenced by factors such as family satisfaction and the severity of the condition [29]. These figures suggest that maintaining engagement in remote rehabilitation remains an important consideration in this field. Many studies in the field of telerehabilitation face limitations such as small sample sizes, which reduce statistical power and external validity, hindering the generalizability of findings [30,31,32]. However, the absence of intervention for the control group may have contributed to a higher dropout rate among these participants. To mitigate this, all families—regardless of group allocation—received, at the end of the study, a comprehensive report detailing the results of the administered questionnaires, which is typically a paid service in clinical settings but was provided free of charge. In addition, families were granted access to home-based activity resources in the form of videos and infographics, aimed at supporting continued rehabilitation efforts.

Although the intervention period in this study was relatively short, previous research involving longer durations—such as seven-week programs—has demonstrated greater impact on family quality of life and children’s social functioning, though not consistently across other developmental domains. High adherence rates are generally associated with telerehabilitation interventions that incorporate caregiver coaching and family-centered models [33,34,35]. Therapist guidance and structured protocols have also been shown to enhance parental engagement. However, challenges such as technological barriers, scheduling conflicts, and caregiver burden—particularly in interventions requiring high parental involvement—can negatively affect adherence [36,37,38]. In this study, families were placed at the center of the intervention, as they were responsible for conducting the evaluations, implementing the activities, and ensuring follow-up—tasks that may be particularly demanding given that this population already tends to report high levels of caregiver burden. These findings suggest that factors like intervention complexity, available support, socioeconomic and technological conditions, and the child’s developmental stage may influence adherence. It may also be worth considering the use of more specific tools to evaluate the impact of mHealth on children’s daily functioning; however, many of these instruments are not yet validated in the Spanish population, which limits their applicability in this context.

In terms of app usability, the SUS has been widely used in digital health due to its short format, its high sensitivity to design issues, and its ability to generate comparable results across contexts and devices. In the field of mHealth apps, this scale is particularly useful, as it allows accurate information to be collected without creating an excessive burden on the user. This result indicates that the app was perceived as generally usable by families, which is critical to ensure adherence and uptake of the intervention. Although the usability rating of the application was in the medium-to-moderate range, it proved sufficient to ensure engagement and continuity in tele-occupational therapy processes, particularly in contexts where families face technological limitations, infrastructural barriers, or follow-up challenges [31,36]. The application was specifically developed for use in rural settings, where resource scarcity and contextual barriers are common. It was also designed to accommodate varying levels of digital literacy, featuring a highly intuitive interface. The intervention materials—such as videos and worksheets—were created using simple and accessible language, in accordance with the principles of cognitive accessibility outlined in ISO 21801-1:2020 [39]. Clinically, this highlights the need for simplified interfaces, hybrid follow-up strategies, and context-sensitive protocols that can be realistically implemented by families with different levels of digital competence and support. The potential of telematic interventions and mHealth applications is particularly relevant in regions where access to continuous and equitable early intervention services is limited. In some autonomous communities in Spain, such as the Principality of Asturias, early care is provided only between the ages of 0 and 3. After this period, responsibility for intervention shifts to the educational system at around age 6, resulting in a significant gap in therapeutic support. This gap is further exacerbated in areas with a high proportion of rural population—such as the one where this study was conducted—where geographical dispersion and reduced availability of specialized services present additional barriers. In this context, digital tools may serve as a valuable complement to existing services, helping to bridge access limitations and support families during critical developmental stages [40]. Consistent with these findings, a systematic review of randomized controlled trials by Mirzakhany et al. (2023) [30] supports the usability and clinical value of tele-occupational therapy for children and adolescents with disabilities, often resulting in increased family satisfaction. Although no significant differences in family quality of life were found in the present study, as in hybrid studies such as Jiménez-Arberas et al. [26,28], this may be due to adherence to this type of intervention.

Among the main limitations of this study are the small sample size and the use of convenience sampling with heterogeneity of diagnostic profiles, which may introduce selection bias and limit the generalizability of the findings. Additionally, the absence of effect size analyses restricts the quantitative interpretation of the magnitude of the observed changes. Moreover, treatment adherence was evaluated through qualitative follow-up indicators and material viewing time; however, future studies should incorporate more systematic and standardized methods to assess adherence more accurately. For future research, it is recommended to expand the sample size to increase statistical power, incorporate effect size analyses to assess the clinical relevance of observed changes, and develop more accurate methods for evaluating and monitoring adherence and family involvement. A possible avenue for future research could involve the development or validation of sensitive and reliable assessment tools with strong psychometric properties, specifically adapted for use in fully remote contexts within the Spanish population. While the PEDI-CAT is one of the most robust and widely used instruments in the literature, there is still limited evidence regarding its application in telerehabilitation settings. Moreover, the inherent heterogeneity of the neurodevelopmental disorder population poses additional challenges to the use of standardized tools for assessing occupational performance, suggesting the need for more tailored approaches in this area. Additionally, it would be beneficial to explore longer-term interventions across diverse profiles and more diagnostically balanced samples, and potential differential effects by diagnostic category should be explored in order to better understand their impact on children’s occupational performance.

Power Considerations and Clinical Significance: This study was underpowered to detect small effect sizes, which may account for the lack of statistical significance in some outcomes. However, significant effects were found in the Responsibility dimension and Total scores, suggesting these impacts were strong enough to surpass power limitations. The observed effect sizes (f = 0.081–0.175), although modest, align with typical behavioral intervention outcomes and may be clinically meaningful, especially in complex behavior change contexts [41]). Notably, improvements in Responsibility (f = 0.175) and Total scores (f = 0.146) indicate meaningful gains in autonomy and adaptive functioning, consistent with the intervention’s goals.

## 5. Conclusions

This pilot study provides preliminary evidence supporting the effectiveness of a tailored mHealth intervention for families of children with neurodevelopmental disorders. Despite being limited by a small sample size and reduced statistical power, the significant improvements observed in Responsibility and Total adaptive behavior scores, along with consistent small-to-medium effect sizes across dimensions, suggest that the intervention holds promise. Furthermore, the rigorous power analysis conducted enhances the methodological transparency of the study and underscores the practical significance of the observed effects.

Importantly, the potential of mHealth as a supportive modality is especially relevant when considering factors such as digital competence and family engagement as part of the inclusion criteria. While the results cannot yet be generalized, they point to telerehabilitation as a valuable complement to in-person services—particularly in contexts where access is restricted due to logistical or economic constraints. A hybrid approach combining remote and face-to-face interventions may help improve functional outcomes in children facing challenges in activities of daily living. These findings lay the groundwork for future, adequately powered trials to more conclusively assess the intervention’s efficacy.

## Figures and Tables

**Table 1:** Descriptive results for the Sensory Profile quadrants, which were used to guide occupational therapy interventions.

Sensory	Behavioral	Seeking	Avoiding	Sensitivity	Registration
50.0	82.0	23.0	38.0	42.0	29.0
40.0	71.0	22.0	26.0	38.0	25.0
54.0	74.0	23.0	36.0	42.0	27.0
52.0	77.0	29.0	31.0	40.0	29.0
35.0	80.0	13.0	38.0	40.0	24.0
34.0	57.0	19.0	19.0	40.0	13.0
50.0	79.0	29.0	34.0	41.0	25.0
19.0	48.0	10.0	21.0	18.0	13.0
29.0	66.0	17.0	25.0	29.0	24.0
24.0	48.0	12.0	23.0	24.0	13.0
16.0	32.0	7.0	10.0	22.0	4.0
33.0	63.0	16.0	29.0	40.0	11.0
31.0	65.0	16.0	31.0	36.0	12.0
M 35.92 (SD = 12.61)	M 64.77 (SD = 15.02)	M 18.15 (SD = 6.85)	M 27.77 (SD = 8.22)	M 34.77 (SD = 8.46)	M19.15 (SD = 8.33)

**Table 2:** PEDI-CAT pre–post-intervention total scores of the experimental and control groups.

Group	Pre-Intervention	Post-Intervention
Experimental Group (Total)	52.7 (2.65)	53.7 (3)
Control Group (Total)	54 (2.28)	54 (2.83)
Dimensions	E.G * 65.6 (2.74) C.G * 65.8 (3.25)	E.G 65.6 (2.51) C.G 65 (2.37)
Mobility	E.G 58.7 (4.97) C.G 62 (3.85)	E.G 60.3 (3.97) CG 62.2 (5.34)
Social ADL	E.G 37.3 (6.52) C.G 43.2 (7.28) E.G 52.7 (2.65) C.G 54 (2.28)	E.G 39.8 (5.33) C.G 41.3 (6.89) E.G 53.7 (3) C.G 54 (2.83)

* E.G (experimental group); C.G (control group).

## Data Availability

Data is unavailable due to privacy and ethical restrictions.

## Abbreviations

The following abbreviations are used in this manuscript:

ADHD	Attention-Deficit/Hyperactivity Disorder
ADL	Activities of Daily Living
ASD	Autism Spectrum Disorder
DSM-5	Diagnostic and Statistical Manual of Mental Disorders
NND	Neurodevelopmental Disorder
